# Lipid Structure Influences the Digestion and Oxidation Behavior of Docosahexaenoic and Eicosapentaenoic Acids in the Simulated Digestion System

**DOI:** 10.1021/acs.jafc.3c02207

**Published:** 2023-06-20

**Authors:** Gabriele Beltrame, Eija Ahonen, Annelie Damerau, Haraldur G. Gudmundsson, Gudmundur G. Haraldsson, Kaisa M. Linderborg

**Affiliations:** † Food Sciences, Department of Life Technologies, University of Turku, FI-20014 Turku, Finland; ‡ Faculty of Pharmaceutical Sciences, University of Iceland, IS-107 Reykjavík, Iceland; § Faculty of Physical Sciences, University of Iceland, IS-107 Reykjavík, Iceland

**Keywords:** DHA, EPA, TAG enantiomers, ethyl ester, in vitro digestion, oxidation

## Abstract

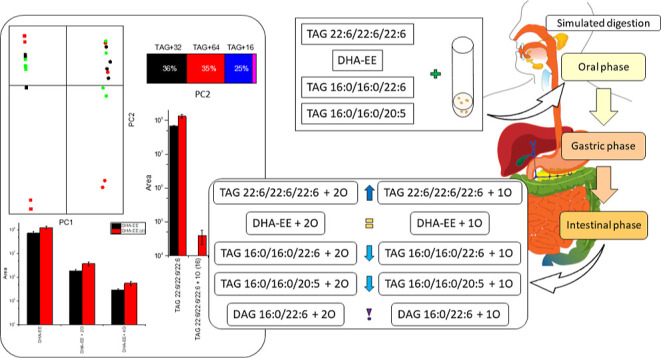

Omega-3 fatty acids such as eicosapentaenoic acid (EPA) and docosahexaenoic acid (DHA) are essential for human health but prone to oxidation. While esterification location is known to influence the stability of omega-3 in triacylglycerols (TAGs) in oxidation trials, their oxidative behavior in the gastrointestinal tract is unknown. Synthesized ABA- and AAB-type TAGs containing DHA and EPA were submitted to static *in vitro* digestion for the first time. Tridocosahexaenoin and DHA as ethyl esters were similarly digested. Digesta were analyzed by gas chromatography, liquid chromatography–mass spectrometry, and nuclear magnetic resonance spectroscopy. Besides the formation of di- and monoacylglycerols, degradation of hydroperoxides was detected in ABA- and AAB-type TAGs, whereas oxygenated species increased in tridocosahexaenoin. Ethyl esters were mainly unaffected. EPA was expectedly less susceptible to oxidation prior to and during the digestion process, particularly in *sn*-2. These results are relevant for the production of tailored omega-3 structures to be used as supplements or ingredients.

## 1 Introduction

Polyunsaturated omega-3 fatty acids (*n*-3 PUFAs), such as eicosapentaenoic acid (EPA) and docosahexaenoic acid (DHA), are essential for human health and development. They are considered crucial in all growth stages, from conception to aging, and are associated with visual development, cognitive health, and protection from cardiovascular diseases.^1^ As components of cell membranes, they affect cell signal transduction, gene expression, cell growth, and apoptosis by changing the fluidity and structure of membrane protein domains.^2^ Moreover, *n*-3 PUFAs act as a cell regulator at the extracellular level with derivatives such as prostaglandins, which act as competitors in the arachidonic acid cascade involved in inflammation.^3^*n*-3 PUFAs are mainly produced in the marine environment, and their main dietary source is seafood. New sources are increasingly sought to counter the inability of fisheries to sustainably supply the minimum required dose of PUFAs to the world population.^4^

The development of products including *n*-3 PUFAs is challenged by their proneness to oxidation. Light, heat, or metal ions can catalyze the abstraction of hydrogen adjacent to double bonds with a lower bond dissociation energy compared to mono- or unsaturated fatty acids. The generated lipid radical reacts with atmospheric oxygen to produce peroxyl radical, which in turn is able to abstract another hydrogen to generate lipid hydroperoxide and another lipid radical. Hydroperoxides homolytically decompose to alkoxy radicals and hydroxy radicals, which, depending on reaction conditions and lipid structure, rearrange or decompose to a plethora of compounds, including aldehydes.^5^ These compounds are responsible for the rancidity and off-flavors, but they are also antinutritional. Triacylglycerol (TAG) hydroperoxides might contribute to atherosclerosis,^6^ while unsaturated aldehydes are considered to be toxic and with mutagenic potential.^7,8^ Protection of PUFAs from oxidation is therefore of utmost importance.

Industrially, PUFA concentrates are commonly produced by chemical/enzymatic transesterification of the natural TAG structure into ethyl esters (EEs) for upper concentration. However, the higher bioavailability of TAG form compared to EE form,^9^ especially from low-fat diets, makes the TAG structure more appealing for product development. The position of PUFAs in the glycerol backbone influences their oxidative stability, with position *sn*-2 being the most protective.^10,11^ Our group also observed that oxidative stability is affected by the lipid structure (TAG or EE).^12^ Therefore, one strategy for the protection of PUFAs from oxidation could be their inclusion in the most stable chemical form, possibly in synthesized TAGs with predetermined distribution of fatty acids in the glycerol backbone.^13^ While most oxidation studies utilized TAGs with randomized PUFA positions or with known *sn*-2 but undistinguished *sn*-1,3 positions,^14^ regio- and even enantiomerically pure TAGs (i.e., TAGs with defined fatty acid positions) are nowadays available.^15,16^ Very recent results from our group^11^ showed that regio- and enantiomerically pure DHA esterified in the *sn*-2 position was the most resistant to oxidation, with or without antioxidants. Moreover, as recently reviewed,^17^ there is evidence pointing to better absorption of DHA by intestinal mucosa when located in position *sn*-2. The same was also observed by our group in a rat feeding trial with regio- and enantiomerically pure TAGs.^18^

Most PUFA oxidation studies focus on production and storage conditions. More recently, attention has also been given to the gastrointestinal tract, which has been characterized to possess pro-oxidant conditions,^19^ due to the oxygen incorporated during mastication, the low pH of the stomach, and the presence of metal ions in food.^20^ In the gastrointestinal tract, gastric lipase starts TAG hydrolysis in the stomach, producing mainly DAGs. As these molecules are amphipathic, they facilitate dispersions of lipids. In the small intestine, lipids are emulsified by bile acids to form micelles, which facilitate hydrolysis to 2-MAGs and free fatty acids. At physiological conditions, micelles are absorbed by the enterocytes, in which TAGs are resynthesized and enter lymphatic circulation.^21^ The hydrolytic activity of lipase during digestion has been correlated to oxidative degradation of oil, although the complexity of the system has made general statements difficult.^22^ Due to ethical reasons and costs, studies on lipid oxidation during digestion generally utilize *in vitro* models. Multiple models are available in the literature, with INFOGEST 2.0^23^ being considered the one with the widest international consensus.

The present study aimed to investigate the oxidation behavior of PUFAs with defined positions on the glycerol backbone in a simulated digestion system. The main aim was to compare structured lipids with DHA and EPA in positions *sn*-1, *sn*-2, or *sn*-3. The study also aimed to compare regio- and enantiomerically pure TAGs with pure DHA in the form of TAG (tridocosahexaenoin) or EE, which are commonly used as *n*-3 PUFA supplements.^24^ Oxidation behavior and digestion products were analyzed together with undigested samples utilizing gas chromatography coupled with flame-ionization detection (GC-FID), liquid chromatography coupled with mass spectrometry (LC–MS), and nuclear magnetic resonance (NMR) spectroscopy.

## 2 Materials and Methods

### 2.1 Chemicals, Samples, and Reagents

All chemical solvents were supplied by Sigma-Aldrich (Saint Louis, MO, USA), unless specified otherwise. All salts used for the simulated digestion were supplied by VWR Chemicals (Leuven, Belgium), unless specified otherwise. Regiopure ABA-type DHA- and EPA-TAGs (*sn*-2) and regio- and enantiopure AAB-type DHA- and EPA-TAGs (*sn*-1, *sn*-3) were synthesized (see Section 2.2). Tridocosahexaenoin (purity > 99%) and ethyl docosahexaenoate (DHA-EE, purity > 99%) were purchased from Larodan (Solna, Sweden). These compounds, once dissolved in hexane, were added with α-tocopherol (0.05% tocopherol/DHA ratio) from Sigma-Aldrich (Buchs, Switzerland) and stored at −80 °C until experiments were performed. For simulated digestion, rabbit gastric lipase (RGE15-500) was obtained from Lipolytic (Marseille, France), while amylase, porcine pepsin, porcine pancreatin, and bile salts were purchased from Sigma-Aldrich (Saint Luis, USA). Potato flour, whey protein concentrate, and wheat bran were supplied by Finnamyl Oy (Kokemäki, Finland), HSNG AB (Solna, Sweden), and Raisio Oy (Raisio, Finland), respectively.

### 2.2 Synthesis of Regiopure and Enantiopure TAGs

Regiopure symmetrically structured ABA-type and regio- and enantiopure asymmetrically structured AAB-type DHA-TAGs and EPA-TAGs were synthesized. The TAGs were synthesized from pure palmitic acid (16:0) and DHA or EPA, all supplied by Larodan (Solna, Sweden). The ABA-type TAGs were synthesized from glycerol by first obtaining 1,3-DAG 16:0/16:0 with lipase CAL-B (Novozymes, Gladsaxe, Denmark), followed by incorporation of DHA or EPA in position *sn*-2 with coupling agent.^15^ On the other hand, the asymmetric AAB-type TAGs were synthesized from *R*- or *S*-solketal (2,2-dimethyl-1,3-dioxolane-4-methanol), after benzyl ether protection and subsequent deprotection of the isopropylidene group and incorporation of palmitic acid into the resulting hydroxyl groups by CAL-B. After deprotection of the benzyl group, DHA or EPA were incorporated with a coupling agent to the remaining hydroxyl group.^25^ The presence of BHT in the final product was verified by NP-HPLC using a Shimadzu Nexera XR LC-30 HPLC instrument (Shimadzu, Kyoto, Japan) equipped with a LiChroCART Superspher Si 60 column (250 × 4 mm; Merck KGaA, Darmstadt, Germany) using hexane with an isocratic flow rate of 0.5 mL/min and UV detection at 287 nm. Concentrations of BHT were calculated by injecting standard solutions of 2.5–100 μg/mL of BHT (Sigma-Aldrich) in hexane. Traces of BHT in the ABA- and AAB-type DHA-TAGs were equalized to 0.018% w/w. No BHT could be detected in the ABA- and AAB-type EPA-TAGs.

### 2.3 Simulated Digestion

#### 2.3.1 Simulated Meal

A simulated meal was prepared for the static *in vitro* digestion experiments. Potato flour, whey protein concentrate, and wheat bran were used as carbohydrate, protein, and fiber sources, respectively. The carbohydrate, protein, and fiber contents were calculated from the composition information provided by the manufacturers. Energy intake was used to calculate the composition of the simulated meal: 52.5% of the energy was derived from carbohydrates, 15% from proteins, and 32.5% from lipids. Fiber was added with the ratio 1 g/79.7 kcal of meal. Potato flour, whey protein, and wheat bran were defatted with a chloroform/methanol 2:1 v/v (although dichloromethane should be preferred) extraction for 48 h. The simulated meal was prepared by mixing the ingredients in adequate ratios and heating at 100 °C for 20 min after the addition of deionized water. Simulated meal, and the lipid component were added separately.^26^ The lipid components were DHA-EE, DHA-TAG, and enantio- and regiopure TAGs containing DHA and EPA in *sn*-1, *sn*-2, or *sn*-3 positions.

#### 2.3.2 Static *In Vitro* Digestion

The simulated *in vitro* digestion was performed according to the INFOGEST 2.0 protocol^23^ with modifications. The whole procedure was carried out in dim light, while digestion itself was carried out in darkness. The simulated saliva fluid, simulated gastric fluid, and the simulated intestinal fluid were prepared according to the protocol. Enzyme solutions in their respective simulated fluids were prepared the day of the experiments. The required aliquots of CaCl_2_·2H_2_O 0.3 M were added to the simulated fluids only prior to enzyme addition. Solutions were preheated to 37 °C before use. The pH was adjusted with HCl and NaOH 1 M solutions. The required aliquots of HCl and NaOH solutions were verified prior to digestions. *Simulated oral phase*: 0.90 g of simulated meal and 30 mg of lipid component were added to 1 mL of simulated saliva fluid, containing alfa-amylase 75 U/mL. The pH of the mixture was adjusted to 7. Chewing process was simulated with a clean glass rod by randomly striking the mixture 32 times. Subsequently, the mixture was incubated for 2 min at 37 °C. Chewing time was excluded from the incubation time. *Simulated gastric phase*. The simulated oral bolus was added to 1 mL of simulated gastric fluid containing pepsin and rabbit gastric lipase to reach enzymatic activity of 2000 U/mL and 60 U/mL, respectively (final chyme volume). After pH adjustment to 3, the mixture was incubated for 2 h at 37 °C. *Simulated intestinal phase*. The simulated gastric chyme was added to 2 mL of simulated intestinal fluid containing pancreatin and bile salts to reach concentrations in the digesta of 6 g/L^27^ and 10 mM, respectively. After pH adjustment to 7, the mixture was incubated for 2 h at 37 °C.

#### 2.3.3 Lipid Extraction

Lipids were extracted immediately after digestion using hexane/isopropanol (2:1, v/v). BHT was added to the extraction solvent at a concentration of 0.05% in order to prevent further oxidations.^28^ Throughout the procedure, samples were kept on ice. The solvent was added to the digestates in a ratio of 1:1 v/v, and tubes were vortexed for 10 s and centrifuged at 1000 rpm for 3 min. The extraction was performed twice. The upper phases were combined and evaporated to dryness with N_2_ at 37 °C. Samples were recovered with 3 mL of chloroform/methanol (1:1, v/v) and stored at −80 °C.

### 2.4 Fatty Acid Analysis

The fatty acids in the samples were methylated for GC analysis. A methanolic hydrochloric acid mixture was prepared by adding cold acetyl chloride (Sigma-Aldrich St. Louis, MO, USA) to cold methanol (1:10, v/v) in ice bath. Heptadecanoic acid (C17:0, Sigma-Aldrich) was used as an internal standard. Aliquots of digestates, after addition of an internal standard, were dried, added to 2 mL of methanolysis mixture, and incubated overnight at 50 °C. Analysis was carried out with a Shimadzu GC-2030 equipped with an autoinjector and an FID detector. Analytical details have been reported elsewhere.^24^

### 2.5 Non-volatile Oxidation Product Analysis

Non-volatile oxidation products were analyzed by Elute UHPLC and Bruker Impact II quadrupole time-of-flight (QTOF) instruments from Bruker Daltonic (Bremen, Germany) and a Phenomenex (Torrance, CA, USA) Kinetex 2.6 μm PS C18 column (100 × 2.1 mm). The method was modified from previous work.^12^ Temperatures for the column oven and autosampler cooler were 30 and 10 °C, respectively. Samples were diluted to 0.01 mg/mL in MeOH/chloroform (1:1, v/v), and 1 μL was injected. A binary solvent system was applied for analyte separation. Solvent A consisted of 95% water (ultrapure from the Purelab Chorus instrument, Elga Veolia, High Wycombe, UK), 5% methanol (Honeywell/Riedel de Haën, Seelze, Germany), and 10 mM ammonium formate (Sigma-Aldrich, Steinheim, Germany). Solvent B comprised of 70% 2-propanol (Honeywell/Riedel de Haën, Seelze, Germany), 30% methanol, 0.1% water, and 10 mM ammonium formate. For the TAG samples, the LC gradient program was as follows: B increased from 60% to 90% in 3 min, to 96.5% in 8 min, to 100% in 0.5 min, held for 2 min, decreased to 60% in 0.5 min, and held for 3 min. For the ethyl ester samples, the following modifications were applied: B increased from 60% to 78% in 3 min, to 85% in 8 min, to 100% in 0.5 min, held for 2.5 min, decreased to 60% in 0.5 min, and held for 4.0 min. The total run time was 18.5 min with a flow rate of 0.3 mL/min. Electrospray ionization (ESI) was applied in the positive mode. The capillary voltage was set to 4.5 kV, and the end plate was offset set to 500 V. Nebulizer gas pressure, drying gas flow rate, and drying gas temperature were 1.5 bar, 4 L/min, and 350 °C, respectively. Auto MS/MS scanning mode from 60 to 1200 *m*/*z* was applied. Internal calibration was performed with sodium formate. The concentration of the samples was equalized before analysis. Peak area values were obtained with Bruker Compass DataAnalysis (Bruker Daltonic, Bremen, Germany).

### 2.6 NMR Spectroscopy

^1^H NMR spectra were collected from undigested lipid components and lipids extracted from digesta. For the analysis, 500 μL of sample was dried with N_2_ flow and recollected to 200 μL of CDCl_3_/DMSO-*d*_6_ (5:1, v/v, previously dried with 4 Å molecular sieves), of which 180 μL was pipetted into 3 mm NMR tubes. Samples were prepared the previous day before the analysis and stored overnight at −20 °C in desiccators. ^1^H NMR spectra were collected at 298 K with a Bruker Avance 600 MHz (Bruker Biospin, Switzerland) equipped with a Prodigy TCI CryoProbe and SampleJet sample changer. Proton spectra were collected with 32 scans, an acquisition time of 4 s, and a relaxation time of 5 s. A selective gradient excitation pulse program (*selgpse*) was applied for region-specific excitation of hydroperoxide (11.5–10.5 ppm) and aldehyde (10–9 ppm) protons. The program had 4 dummy scans and 128 scans, with an acquisition time of 2.7 s and a relaxation time of 5 s. The 180-degree shaped pulse had a length of 1566.15 s.^12^ NMR data were processed with TopSpin 4.0.7 (Bruker, MA, USA).

### 2.7 Statistical Analysis

The statistical analysis was performed with RStudio.^29^ Shapiro–Wilk and Bartlett tests (*stats* package) were used to assess the normality of the data and the homogeneity of the data variance. A t-test was performed with the function *t_test* (*rstatix* package). Analysis of variance was performed with the functions *aov* and *TukeyHSD* (*stats* package) for the ANOVA test and Tukey’s posthoc test or *tamhaneT2Test* (*PMCMRplus* package) for TamhaneT2 posthoc test. The statistical significance of differences among samples was considered at a confidence level of 95% (*p* < 0.05). Principal component analysis of the HPLC-qTOF data was performed with the *PCA* function (*FactoMineR* package) of RStudio. The most important loadings of the obtained model were selected according to their contribution to each computed principal component, using as cutoff the reverse number of compounds used to compute the PCA model.

## 3 Results and Discussion

### 3.1 Fatty Acid Ratio and Fatty Acid Composition

**Figure 1:**
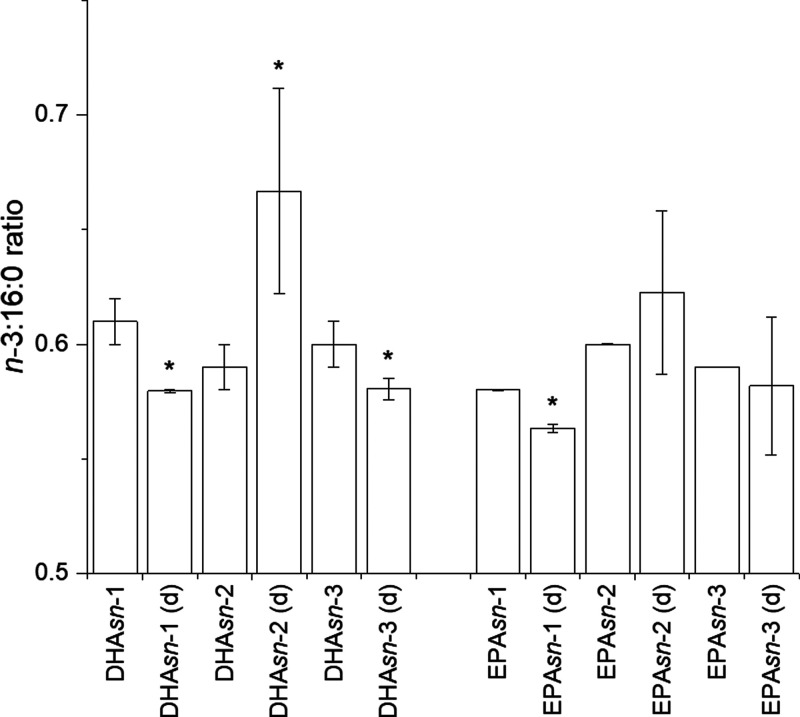
PUFA (DHA and EPA) to 16:0 weight ratio of synthesized ABA- and AAB-type TAGs containing DHA and EPA in either *sn*-1, *sn*-2, or *sn*-3 positions and 16:0 in the remaining positions prior to and after digestion. The letter (d) marks the digestates. Values are average ± standard deviation (*n* = 2). The symbol marks statistically significant difference (*p* < 0.05).

As PUFAs are consumed during the oxidation process, the decrease in the PUFA:16:0 ratio can be utilized as an oxidation indicator for regio- and enantiopure lipids. The ratios were calculated utilizing the fatty acid composition of digestates obtained from the gas-chromatographic analysis (Figure 1). The fatty acid composition of undigested and digested samples is reported in Supporting Information Figure S1. Prior to digestion, the PUFA:16:0 ratio was about 0.60 for all regio- and enantiopure lipids. After digestion, there was a statistically significant (*p* < 0.05) decrease in ratio for DHA*sn*-1 (0.58), DHA*sn*-3 (0.58), and EPA*sn*-1 (0.56) (Figure 1). In the case of DHA*sn*-2, the average ratio was higher after digestion (0.67 against 0.59, *p* < 0.05), and the same was observed for EPA*sn*-2, although with a lack of statistical significance (0.64 against 0.60, *p* > 0.05). Finally, EPA*sn*-3 had a decrease in ratio after digestion but with a high deviation (0.58 against 0.59, *p* > 0.05). Auto-oxidation experiments have assigned position *sn*-2 a protective effect against oxidation.^11,30^ In addition to this, the rabbit gastric lipase utilized in the gastric phase is stereoselective for position *sn*-3,^31^ therefore determining a release of 16:0 from this position in case of DHA*sn*-2 and EPA*sn*-2. Also, this enzyme has lower affinity for long-chain and unsaturated fatty acids.^32^ The high deviation for EPA*sn*-3 could be explained by the hydrolytic effect of pancreatic lipase, which is less selective for position *sn*-3 and also effects position *sn*-1. On the other hand, the difference in the PUFA:16:0 ratio in these two digestates could also be explained by the higher resistance to oxidation by EPA compared to DHA in similar chemical structures.^31,33^ The gas chromatographic analysis of DHA-TAG and DHA-EE digestates showed a decrease in DHA compared to the standard compound of 8.9 ± 0.2 and 9.2 ± 0.7%, respectively. It can be deduced that the PUFA amounts in digestates would be influenced by different factors, such as oxidation (and protection from) and lipase activity.

### 3.2 Non-volatile Digestion and Oxidation Compounds

#### 3.2.1 Identification of Undigested Compounds and Digestion Products

**Figure 2:**
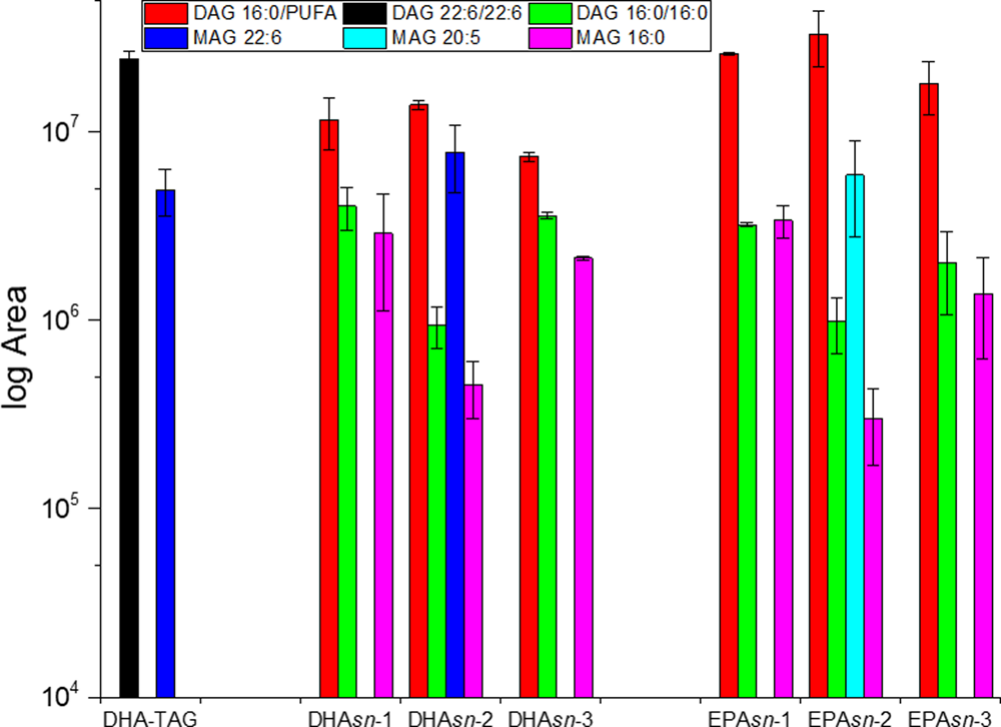
Peak area values (in logarithmic scale) of digestion products identified in HPLC-qTOF chromatograms of digested docosahexaenoin (DHA-TAG) and AAB- and ABA-type TAGs containing DHA and EPA. For their respective classes of compounds, DAG PUFA indicates DAG 16:0/22:6 and DAG 16:0/20:5. Values are average ± standard deviation (*n* = 3 for DHA-EE and DHA-TAG; for others, *n* = 2).

**Figure 3:**
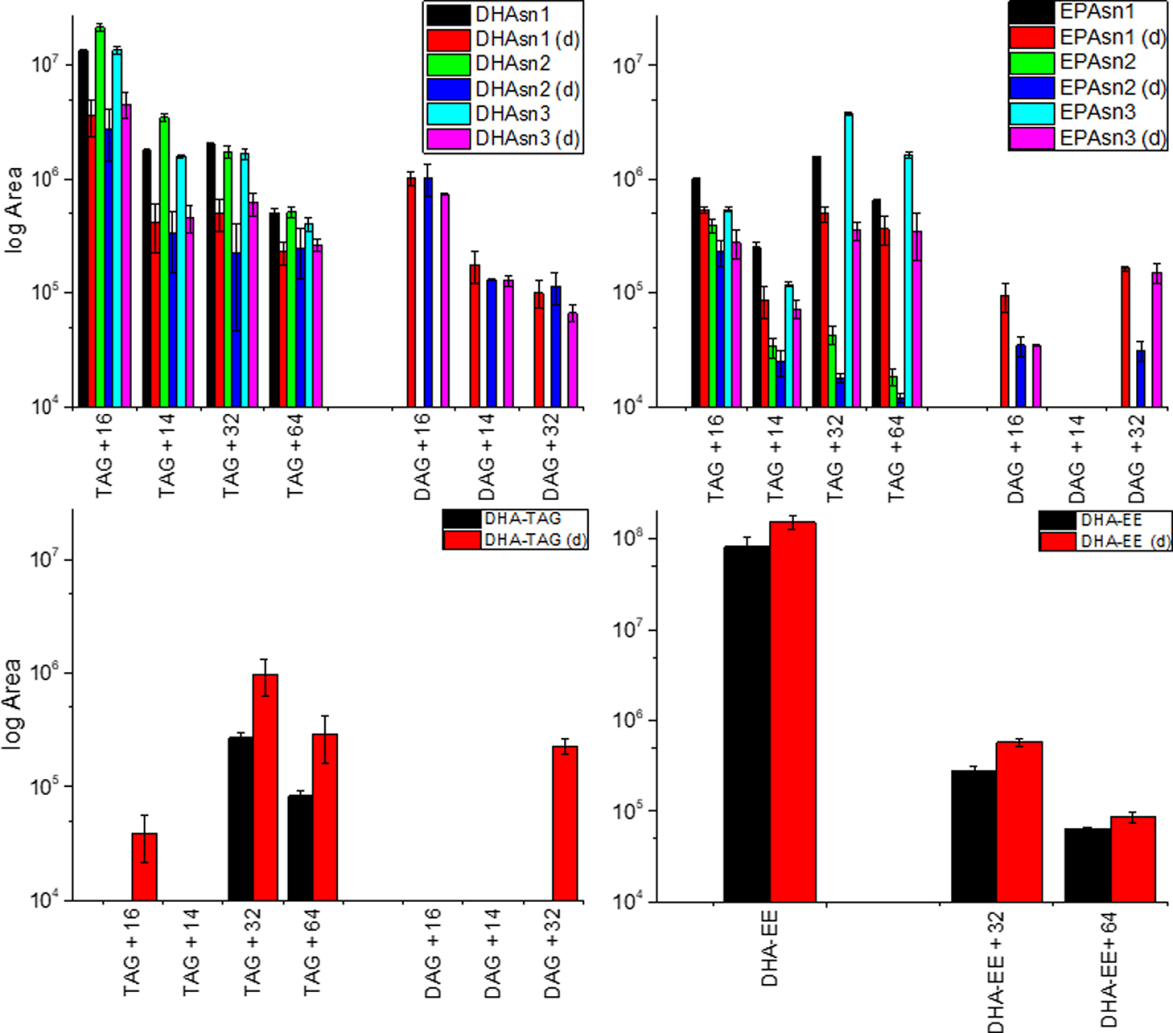
Peak area values (in the logarithmic scale) of oxidated compounds identified in HPLC-qTOF chromatograms of undigested and digested AAB- and ABA-type TAGs, docosahexaenoin (DHA-TAG), and ethyl docosahexaenoate (DHA-EE). Digestates are marked with (d). Values are average ± standard deviation (*n* = 3 for DHA-EE and DHA-TAG; for others, *n* = 2).

Compound peak area data was obtained from the extracted ion chromatograms of undigested standards and digestate extracts. Compounds were identified as ammonium and sodium adducts. Table 1 reports mass values (*m*/*z*) for the identified compounds. Ammonium adducts were the most abundant for unoxidized TAGs and DHA-EE, whereas oxidation products, DAGs (except DAG 16:0/22:6) and MAGs, had the sodium adduct as the most abundant. The integration results are reported in Figures 2 and 3. Details for the identification of undigested lipids, digestion compounds, and primary oxidation products are reported in Supporting Information Text S1.

**Table 1:** Compounds and Their Ammonium [M + 18]^+^ and Sodium [M + 23]^+^ Adduct Masses (*m*/*z*) Identified in HPLC-qTOF Chromatograms, together with Their Retention Times and Fragments Used for Identification^a^

	*m*/*z*	[M + 18]^+^	[M + 23]^+^	r.t. (min)	main fragments		*m*/*z*	[M + 18]^+^	[M + 23]^+^	r.t. (min)	main fragments
DHA regio-/enantiopure						EPA regio-/enantiopure					
16:0/16:0/22:6	878.7	**896.7**	901.7	9.5	551.5, 623.5, 313.3, 311.3, 239.2	16:0/16:0/20:5	852.7	**870.7**	875.7	8.8	551.5, 597.5, 313.3
16:0/16:0/22:6 + 1O (16)	894.7	912.7	**917.7**	7.8	367.2, 661.5, 551.5	16:0/16:0/20:5 + 1O (16)	868.7	886.7	**891.7**	7.6	617.5, 341.2, 551.5
16:0/16:0/22:6 + 1O (14)	892.7	910.7	**915.7**	7.2	365.2, 659.5, 551.5	16:0/16:0/20:5 + 1O (14)	866.7	884.7	**889.7**	7.0	
16:0/16:0/22:6 + 2O	910.7	928.7	**933.7**	6.8	887.7, 806.6, 847.7, 383.2	16:0/16:0/20:5 + 2O	884.7	902.7	**907.7**	6.6	781.5, 357.2, 551.5
16:0/16:0/22:6 + 4O	942.7	960.7	**965.7**	5.9		16:0/16:0/20:5 + 4O	916.7	934.7	**939.7**	5.7	
16:0/16:0	568.5	**586.5**	591.5	5.3	313.3, 551.5, 339.3	16:0/16:0	568.5	**586.6**	591.5	5.3	313.3, 551.5, 339.3
16:0/22:6	640.5	**658.5**	663.5	5.1	313.3, 385.3, 641.5	16:0/20:5	614.5	**632.5**	637.5	4.9	313.3, 359.3, 285.2
16:0/22:6 + 1O (16)	656.5	674.5	**679.5**	4.6	367.2, 661.5, 313.3	16:0/20:5 + 1O (16)	630.5	648.5	**653.5**	4.5	
16:0/22:6 + 1O (14)	654.5	672.5	**677.5**	4.4							
16:0/22:6 + 2O	672.5	690.5	**695.5**	4.3		16:0/20:5 + 2O	646.5	664.5	**669.5**	4.2	
MAG 16:0	330.3	348.3	**353.3**	2.9	335.3, 226.2, 263.2	MAG 16:0	330.3	**348.3**	353.3	2.9	335.3, 226.2, 263.2
MAG 22:6	402.3	**420.3**	425.3	2.7	311.2, 385.3, 403.3, 293.2	MAG 20:5	376.3	**394.3**	399.3	2.3	267.2, 285.2, 303.2
DHA-TAG						DHA-EE					
22:6/22:6/22:6	1022.7	**1040.7**	1045.7	8.2	695.5, 385.3	DHA-EE	356.3	**374.3**	379.3	4.9	311.2, 293.2
22:6/22:6/22:6 + 1O (16)	1038.7	1056.7	**1061.7**	7							
22:6/22:6/22:6 + 2O	1054.7	1072.7	**1077.7**	6.2	383.2, 950.6, 991.6	M + 2O	388.3	406.3	**411.3**	3.2	365.2, 285.1, 245.1, 393.2
22:6/22:6/22:6 + 4O	1086.7	1104.7	**1109.7**	5.4		M + 4O	420.3	438.3	**443.3**	1.2	
22:6/22:6	712.5	**730.5**	735.5	4.9	385.3, 311.2, 293.2						
22:6/22:6 + 2O	744.5	762.5	**767.5**	4.2							
MAG 22:6	402.3	420.3	**425.3**	2.7	239.2, 308.8						

a Mass values in bold represent ion currents used for identification.

#### 3.2.2 Influence of Lipid Structure on the Formation of Digestion Products

At our experimental conditions, no statistically significant decrease in the area of AAB- and ABA-type TAGs was observed, whereas, in the case of DHA-TAG and DHA-EE, the area of undigested molecules increased. This could be attributed to a higher ionizability of the molecules, likely due to residual salts in the digestate extracts. In terms of DAGs, the comparison of AAB- and ABA-type TAGs showed that DHA*sn*-2 produced more (*p* = 0.003) DAG 16:0/22:6 than DHA*sn*-3. Despite the higher affinity of lipase for *sn*-3 than *sn*-1, production of DAG 16:0/22:6 from DHA*sn*-1 and DHA*sn*-3 had no difference. EPA*sn*-1 produced DAG 16:0/20:5 significantly more than DHA-containing TAGs (*p* < 0.05), but no more than EPA*sn*-3. The peak area values of DAG 16:0/PUFA and DAG 16:0/16:0 (Figure 2) were generally higher for EPA-containing TAG compared to DHA, indicating higher lipolysis. This could be explained by the higher steric hindrance of 22:6 compared to 20:5,^34^ especially regarding the *sn*-3 position, which is preferentially hydrolyzed, and by the decrease in lipolytic activity of rabbit gastric lipase with the increase in carbon chain length.^32^ DAG 16:0/16:0 (Figure 2) could be either a reminder of TAG synthesis or a hydrolysis product of PUFA*sn*-3 (by both lipases) and PUFA*sn*-1 (by pancreatic lipase). The observed increase of DAG 16:0/16:0 after digestion of DHA*sn*-2 and EPA*sn*-2 had no statistical significance (*p* > 0.1 for both). At the same time, there was no significant difference between the areas of DAG 16:0/16:0 after digestion of *sn*-1 and *sn*-3 TAGs. A similar trend was observed with MAG 16:0, found solely in digestates as a hydrolytic product of either DAG 16:0/16:0 or DAG 16:0/PUFA, possibly indicating that the steric hindrance of DAGs generated by PUFAs was lower than in TAGs. The presence of MAG 16:0 in *sn*-2 digestates can be explained as the hydrolytic product of remainder DAG 16:0/16:0 since neither lipase has affinity for position *sn*-2. Moreover, its presence could be explained by the formation, due to acyl migration, of 1,3-DAGs from 1,2-DAGs and their consequent hydrolysis to MAG. However, the absence of MAG 22:6 and MAG 20:5 in the respective *sn*-1 and *sn*-3 digestates indicated acyl migration was a marginal phenomenon under our experimental conditions. The production of MAG PUFA has biological relevance, as 2-MAGs are well absorbed by intestinal mucosa and grant higher bioavailability of PUFAs compared to 1- or 3-MAGs.^17^

Digestion of tridocosahexaenoin produced, as expected, DAG 22:6/22:6 and MAG 22:6 (Figure 2). The area of the latter was statistically similar to the areas of MAG 22:6 and MAG 20:5 originating from DHA*sn*-2 and EPA*sn*-2 (*p* > 0.05). Therefore, despite the steric hindrance of DHA, hydrolysis of position *sn*-3 was overall similar between DHA-TAG and AAB- and ABA-type EPA-TAGs. This is possibly due to the absence of 16:0 in DHA-TAG. At the same time, the area of DAG 22:6/22:6 differed only from DHA*sn*-1 (*p* < 0.05) and DHA*sn*-3 (*p* < 0.01), suggesting DAGs were less hindered than TAGs. Our analytical conditions could not unambiguously assign free fatty acids, and no digestion products of DHA-EE were observed.

#### 3.2.3 Influence of Lipid Structure on Oxidized Compounds during Digestion

**Figure 4:**
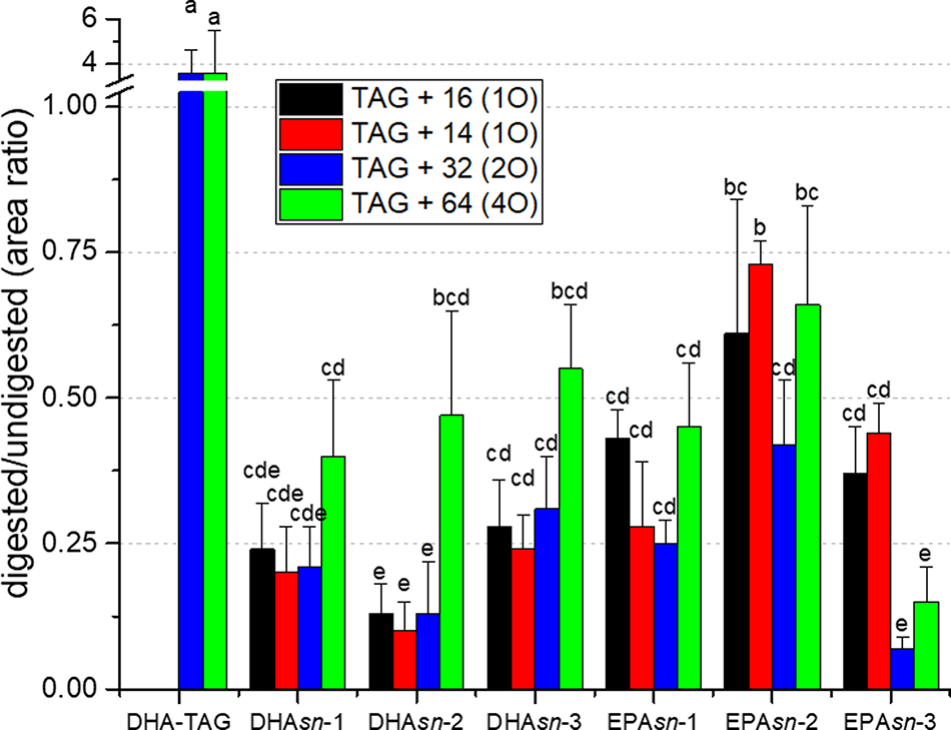
Area ratio of oxygenated structural lipids after and prior to digestion. Values are average ± standard deviation (*n* = 3 for DHA-TAG; for others, *n* = 2). Different letters mark statistically significant difference (*p* < 0.05).

According to our results, simulated digestion under our experimental conditions determined a reduction in oxygenated AAB- and ABA-type structures and the appearance of oxygenated digestion products. On the other hand, an increase in oxygenated species was observed for DHA-TAG and DHA-EE (Figure 3). Compared to the other samples, EPA*sn*-2 had the lowest signals for these molecules, indicating the protective effect of position *sn*-2 and higher stability of this PUFA, as could be expected. Also, only for EPA*sn*-2, the decreases in TAG + 16 (1O), TAG + 14 (1O), and TAG + 32 (2O) lacked statistical significance (0.08 < *p* < 0.9). Conversely, the decrease in TAG + 64 lacked statistical significance for DHA*sn*-2, DHA*sn*-3, and EPA*sn*-2. The area ratio of oxygenated TAGs after and prior to digestion is reported in Figure 4. For EPA*sn*-2, the area of most oxygenated species decreased only about 25% (ratio 0.75), except TAG + 32, which decreased to a ratio of 0.42. On the other hand, DHA*sn*-2 had the highest decreases (ratios 0.16, 0.13, and 0.19 for TAG + 16, TAG + 14, and TAG + 32, respectively) except for TAG + 64, whose decrease had no significant difference (*p* > 0.05) with other decreases, as observed in Figure 3. A ratio of 0.5 (a decrease of 50%) was observed for TAG + 64 species of about all AAB- and ABA-type structures except EPA*sn*-3. Overall, EPA*sn*-2 had the lowest decrease in oxygenated species, while DHA*sn*-2 had the highest, without taking into account the seemingly scantly affected TAG + 64 (Figure 4). In the case of EPA*sn*-2, DAG 16:0/20:5 + 32 had about 1.75 times the area of the respective TAG + 32 (Supporting Information Figure S3). In this case, it can be deduced that the loss of 16:0 decreased the protective effect of *sn*-2 and that new oxidized species were formed. According to our results, there was no correspondence between the loss of oxygenated TAG and the area of oxygenated DAG of regio- and enantiopure compounds, as the areas of the latter corresponded to only 3–14% of the losses. Therefore, most of the losses of TAG + 16, TAG + 14 (for DHA), and TAG + 32 compounds were ascribable to further degradation. Marquez-Ruiz et al. described hydroxy and keto fatty acids as relatively stable in digestion conditions, but their experiments were conducted with a saturated fatty acid. Conversely, their degradation of hydroperoxidated and epoxidated methyl linoleate was, despite the difference in chemical structure, in general agreement with our results.^35^

Differently from regio- and enantiopure TAGs, oxygenated DHA-TAG increased after digestion (Figure 3). Moreover, the addition of one oxygen was observed only in digestates, but noticeably only in TAG species. The ratio between DAG 22:6/22:6 + 32 and TAG 22:6/22:6/22:6 + 32 and the ratio between DAG 22:6/22:6 and TAG 22:6/22:6/22:6 had no significant difference (*p* > 0.05). As pancreatic lipase is proven to act also on oxidized TAGs,^36^ it could be concluded that, while DAG 16:0/PUFA + 32 was mainly the oxidation product of DAG 16:0/PUFA, DAG 22:6/22:6 + 32 arose from lipolysis of the oxidized TAG. It is believed that TAG lipolysis rate is not affected by peroxidation under gastrointestinal conditions^37^ and changes in lipase activity are usually observed after oxidation at cooking temperatures^38^ or prolonged oxidation^39^ experiments. Different from TAGs, DHA-EE was the sole lipid unaffected by digestion, since the area increase was identical for all the species (Figure 3) and therefore ascribable solely to a higher ionizability. This finding is in agreement with previous results from Song and co-workers^40^ and our group,^12^ which have shown, although under different oxidative conditions, the higher stability of DHA-EE compared to DHA-TAG when oxidized in the presence of α-tocopherol. However, Sullivan and co-workers have reported higher oxidation rates for EE of DHA and EPA in comparison with their TAG forms at 30 °C. Further studies monitoring primary and secondary oxidation products could address this discrepancy.^100^

#### 3.2.4 PCA of HPLC-qTOF Data

**Figure 5:**
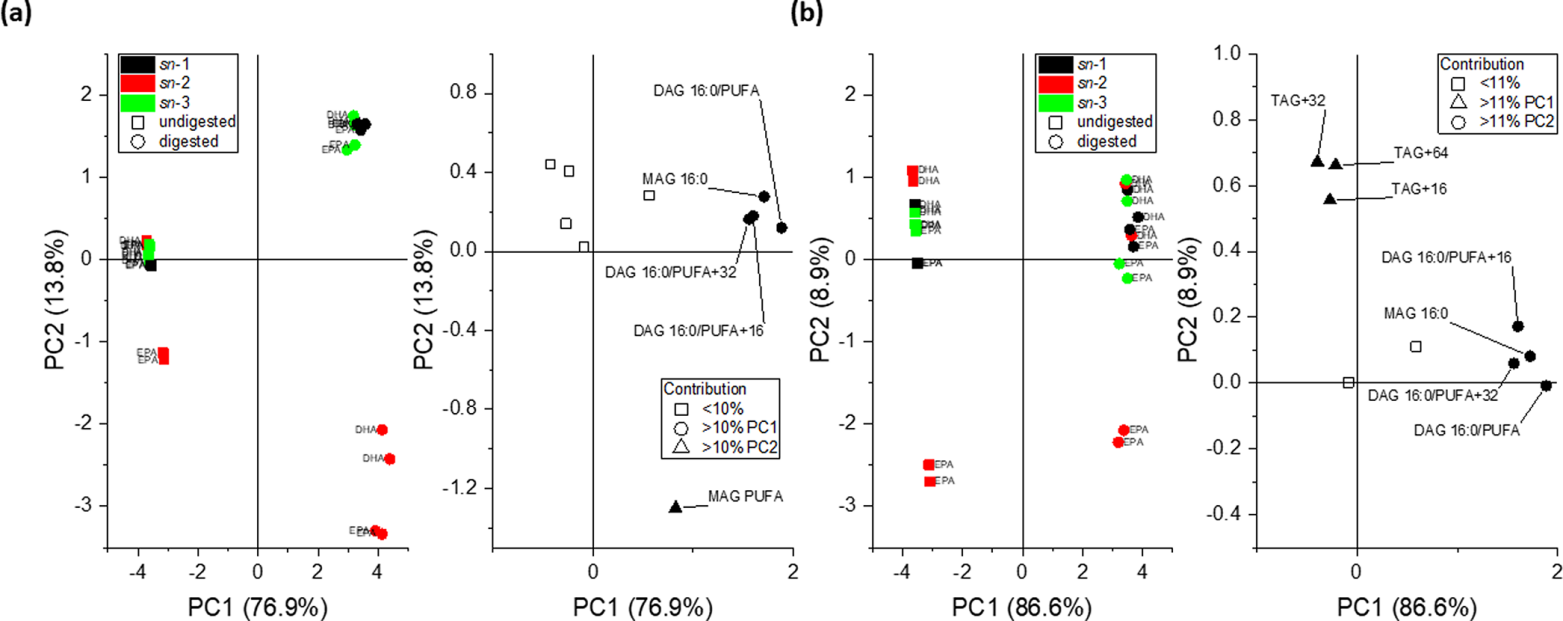
Principal component analysis of HPLC-qTOF data of structured lipids. Model computed with all the unoxygenated and oxygenated species in common between DHA- and EPA-structured lipids (a). Model computed with all the species in common, excluding MAG PUFA (b).

Scores and loading plots of the principal component analysis (PCA) of the HPLC-qTOF data of structured lipids are reported in Figure 5. The first principal component separated digestates from undigested samples and represented more than 75% of the data variance. In the first model (Figure 5a), digestates of EPA*sn*-2 and DHA*sn*-2 grouped together in the lower right quadrant of the score plot, while digestates from *sn*-1 and *sn*-3 grouped together in the upper right quadrant. Undigested samples were all grouped together except for EPA*sn*-2. Therefore, the second principal component discriminated *sn*-2-structured lipids from the others (14% of the variance). The compounds more responsible for the discrimination (contribution higher than 10%) were DAG PUFA, MAG 16:0, DAG PUFA + 16, and DAG PUFA + 32 for PC1 and MAG PUFA for PC2. As MAG PUFA is found only in *sn*-2 digestates (and hence its relevance for discrimination), a PCA model was computed without this compound (Figure 5b). In the second model, digestates were discriminated again according to DAG PUFA, MAG 16:0, DAG PUFA + 16, and DAG PUFA + 32. On the other hand, the discrimination on the second principal component was determined by oxidized TAGs (TAG + 16, TAG + 32, and TAG + 64). Score values on the second principal component are highlighted in Supporting Information Figure S6. As position on PC2 was determined by oxidation status prior to and after digestion, EPA*sn*-2 was positioned farthest from the other samples. However, while prior to digestion, EPA*sn*-1 was the second-farthest sample, EPA*sn*-2 had lower scores after digestion, while the score of EPA*sn*-1 increased. This positioning hinted at a correlation trend between EPA position and amounts of oxidized TAGs after digestion: *sn*-2 < *sn*-3 < *sn*-1. The protective effect of position *sn*-2 has already been reported and has been attributed to steric hindrance.^11,30^ Moreover, this protective effect has also been observed in emulsion systems.^14^ Differences between *sn*-1 and *sn*-3 have been scarcely addressed in the literature, as these compounds are difficult to obtain. In our group, slightly higher stability of *sn*-3 compared to *sn*-1 was observed during auto-oxidation of DHA-containing regio- and enantiopure TAGs in the absence of antioxidants, while the opposite was observed in their presence.^11^ The lower correlation of EPA*sn*-3 with oxidized TAGs after digestion indicated a higher reduction in their amounts compared to EPA*sn*-1 as their positioning on PC2 switched. Therefore, the PCA hinted at a higher stability at digestion conditions of oxygenated PUFA in position *sn*-1, meaning that EPA*sn*-1 hydroperoxides could be more stable than in EPA*sn*-3. However, it is also possible that EPA*sn*-1 hydroperoxides degraded and new species formed at a higher rate than EPA*sn*-3, which would indicate higher stability of EPA*sn*-3 compared to EPA*sn*-1. This hypothesis would be supported by Larsson et al., who reported little change in hydroperoxide amounts in cod oil after simulated digestion but a net increase in secondary oxidation products.^41^ Nevertheless, further experiments are being undertaken by our group to verify the stability of EPA-containing regio- and enantiopure TAGs during auto-oxidation. More studies on the detailed kinetic profile of primary oxidation products at simulated digestion are also required.

### 3.3 ^1^H NMR Spectroscopy

The collected ^1^H NMR spectra are reported in Supporting Information Figures S4 and S5. All the TAG samples showed the typical signal pattern, with the *sn*-1/3 and *sn*-2 position signals around 4.14, 4.29, and 5.24 ppm, respectively. Conversely, the DHA-EE spectrum had the methylene signal of the ethyl chain at 4.12 ppm. In ABA and AAB structures, the terminal methyl signal of 16:0 was observed at 0.88 ppm, while the terminal methyl signal of the ethyl chain of DHA-EE was at 1.25 ppm. In DHA-TAG, DHA-EE, and structured lipids, the terminal methyl signal of PUFA was at 0.97 ppm. After digestion of DHA-EE, a small but significant decrease in the ratio between ethyl −CH_2_– and terminal −CH_3_ of DHA was observed (12.2%, *p* = 0.007), which can be attributed to the hydrolytic activity of lipase. Lower hydrolytic rates of ethyl esters, compared to TAGs, have also been observed by others^42^ and have been considered an explanation for the lower bioavailability of PUFAs in this chemical form.^9^ On the other hand, after digestion of TAGs, DAG and MAG signals were observed. The signals were ascribed to 1,2-DAG (5.07, 4.35, and 3.64 ppm) and 2-MAG (4.88 and 3.70 ppm). In digestates, the quartet at 4.14 ppm was overlapping with another signal, which could either be due to 1,2-DAG or 1,3-DAG. The latter, if present, could not be clearly assigned as it typically produces a broad multiplet at 4.04–4.21 ppm.^15^

The ^1^H NMR spectra obtained after selective gradient excitation of the hydroperoxide region are reported in Supporting Information Figure S7. Regio- and enantiopure TAGs with DHA and DHA-TAG had similar peroxide proton signals, which were observed at 10.93, 11.04, and 11.07 ppm. The first signal can be assigned to a linear structure, the other two to cyclic forms, or to hydroperoxides positioned farther from the terminus of the fatty acid chain.^43^ Regio- and enantiopure TAGs with EPA had an additional proton peak at 11.01 ppm, which could belong either to a linear or cyclical structure.^43^ Among all samples, EPA*sn*-2 and DHA-EE were the sole proton spectra completely lacking hydroperoxide signals, confirming the lower degree of oxidation observed with HPLC-qTOF (Section 3.2 and Figure 2b). After digestion, hydroperoxide proton signals disappeared from the spectra. For AAB- and ABA-type structures, this confirmed the reduction in oxygenated species observed with HPLC-qTOF (Figure 2b). The disappearance of hydroperoxide protons in DHA-TAG digestates could indicate that hydroperoxides are further degraded to hydroxides or epoxides. Such a pathway would be confirmed by the appearance of TAG + 16 after digestion of DHA-TAG (Figure 2b). Peroxidated bisallylic systems are considered to produce a hydroxy moiety after radical scission of the O–O bond and quenching of the generated alkoxy radical. Alternatively, the radical would react with the proximal unsaturated carbon, generating an epoxide.^44,45^ Scission radicals further continue the oxidation pathway to secondary products such as aldehydes, which are reactive with proteins and DNA. The most toxic ones are malondialdehyde and 4-hydroxy-2-alkenals.^7^ The earlier originates from cyclic hydroperoxides, the latter from further oxidation of hydroxy-PUFA.^5^ Different factors, such as micelle inclusion and steric hindrance, might have impeded the radical quenching activity of α-tocopherol in DHA-TAG and rather favored oxidation, while DHA-EE was more stable and lacked hydroperoxide protons even prior to digestion. In the case of ABA and AAB-type TAGs, no antioxidant was added (besides BHT trace normalization) and, therefore, oxidative degradation was possibly ruled by steric hindrance (i.e., position of the PUFA). At the same time, the ratio between PUFA and 16:0 (Figure 1) indicated a lower presence of abstractable protons in the structure, compared to DHA-TAG.

### 3.4 Considerations for Oxidation in the Gastrointestinal Tract

According to the recent work of Takahashi et al., when partially hydroperoxidized or hydroxylated TAGs were introduced into the rat stomach, these compounds were also detected in the lymph, while these compounds were absent when no oxidized molecule was introduced. At the same time, deuterium labeling indicated no absorption of hydroperoxidized or hydroxylated TAGs. Therefore, oxidation damage propagated in the gastrointestinal tract. The same authors reported the reduction of hydroperoxidized fatty acids to hydroxy species not only in vivo but also ex vivo with rat small intestine mucosa homogenate.^46^ This reduction has also been observed in Caco-2 cell lines by other authors.^47^

While there is general agreement on the pro-oxidant conditions of the gastrointestinal tract, the effect of lipolysis on lipid oxidation is less understood. Larsson et al. were among the first ones to observe an increase, although little, in primary and secondary oxidation products of cod oil at simulated digestion conditions after addition of fungal lipase (although now considered inadequate to represent human lipolysis^23^). They also noticed higher amounts of secondary oxidation products after the addition of bile salts, pointing to the importance of micelles for the oxidation pathway.^41^ In addition, Tullberg et al. connected the marked increase in 4-hydroxy-nonenal, originating from *n*-6 PUFAs, in cod liver oil digestates to the presence of rabbit gastric lipase. On the other hand, the presence of gastric lipase had no effect on the marker of *n*-3 PUFAs degradation, 4-hydroxy-hexenal, during the gastric phase, while the marker increased during the intestinal phase.^28^ This was in agreement with the positional distribution of fatty acids in cod liver oil, which had 18:2*n*-6 mainly in *sn*-1,3 positions and DHA solely in *sn*-2 position.^48^ Compared to the previous studies, the present work demonstrated that primary oxidation products arose during digestion, and the extent depended on the lipid structure. They degraded to hydroxides or epoxides in the simulated gastrointestinal tract medium. Our work excluded the necessity of epithelial cells for propagation, pointing to the importance of micelles. The decrease in oxidized molecules during simulated digestion conditions can be ascribed to their chemical instability, as mentioned previously. This degradation would continue oxidation cascade in the gastrointestinal lumen or in micelles, while epoxides and ketones can in addition covalently bind proteins. Liberation of oxidized fatty acids from position *sn*-3 would likely facilitate this process. At the same time, hydrolysis of position *sn*-3 decreases the protection of PUFA in position *sn*-2. While epoxides are reactive and expected to degrade prior to absorption, hydroxides, on the other hand, are proven to be absorbed during digestion,^49^ hence potentially degrading at other sites. Therefore, hydroperoxidation can exert damage locally or after absorption. Further research on the improvement of simulated digestion conditions (amount of required pancreatin or presence of epitelial cells) to more accurately represent the small intestine is therefore required to better understand the dynamics of hydroperoxide degradation.

In conclusion, the present study investigated the simulated digestion of DHA-EE, DHA-TAG, and AAB- and ABA-type enantio- and regiopure TAGs containing DHA and EPA. As previously observed in auto-oxidation trial, DHA-EE showed higher oxidative stability than DHA-TAG in simulated digestion conditions. While DHA-TAG showed an increase in oxidation species during simulated digestion, it generated MAG 22:6, which would grant absorption of PUFA, whereas DHA-EE was only partially hydrolyzed at our digestion conditions. All AAB- and ABA-type TAGs generated MAGs and DAGs, the latter also present as oxidized species. Simulated digestion determined a decrease in oxidized TAGs. In general, EPA was more resistant to oxidation and less resistant to digestion compared to DHA. While the higher stability of PUFAs in *sn*-2 has been reported previously, at simulated digestion conditions, only EPA*sn*-2 was clearly the most stable. In addition, our results hint at a higher degradation rate of EPA*sn*-1 hydroperoxides compared to EPA*sn*-3. Finally, only DHA*sn*-2 and EPA*sn*-2 generated MAG 22:6 and MAG 20:5, which are more efficiently absorbed than the corresponding free fatty acids, giving relevance to our finding regarding bioavailability.

## Supporting Information Available

Identification of undigested compounds and of digestion and oxidation products; fatty acid composition of DHA-containing structured lipids and EPA-containing ABA and AAB structures, DHA-TAG, and DHA-EE; HPLC-qTOF ion currents of DHA*sn*-2 before and after digestion; ratio between oxygenated DAGs and TAGs in digestates of ABA- and AAB-type TAGs and DHA-TAG; ^1^H NMR spectra of ABA and AAB structures, DHA-EE, and DHA-TAG prior to and after digestion; average score values on the second principal component of the PCA model computed with HPLC-qTOF data of structural lipids after removal of MAG PUFA; and selective gradient excitation ^1^H NMR spectra of ABA- and AAB-structures, DHA-EE, and DHA-TAG prior to and after digestion (PDF)

## Abbreviations

ANOVA: analysis of variance
BHT: dibutylhydroxytoluene
CDCl_3_: deuterated chloroform
DAG: diacylglycerol
DHA: docosahexaenoic acid
DMAP: 4-dimethylaminopyridine
DMSO-*d*_6_: deuterated dimethylsulfoxide
DCl: 1-ethyl-3-(3-dimethylaminopropyl)carbodiimide
EE: ethyl ester
EPA: eicosapentaenoic acid
GC-FID: gas chromatography coupled with flame ionization detection
HPLC-qTOF: high-performance liquid chromatography with triple quadrupole-time-of-flight detection
MAG: monoacylglycerol
NMR: nuclear magnetic resonance
PCA: principal component analysis
PC1(2): principal component 1(2)
PUFA: polyunsaturated fatty acid
TAG: triacylglycerol
