# Supplementary material for: Lipid Structure Influences the Digestion and Oxidation Behavior of Docosahexaenoic and Eicosapentaenoic Acids in the Simulated Digestion System
Source: J Agric Food Chem. 2023 Jun 20;71(26):10087–96. doi: 10.1021/acs.jafc.3c02207 (PMC10326875; doi:10.1021/acs.jafc.3c02207)
Supplement: Supplementary file 1 — jf3c02207_si_001.pdf [file jf3c02207_si_001.pdf]

Supporting Information

Lipid structure influences the digestion and oxidation behavior of docosahexaenoic and eicosapentaenoic acids in simulated digestion system

Gabriele Beltrame[†], Eija Ahonen[†], Annelie Damerau[†], Haraldur G. Gudmundsson[‡], Gudmundur G. Haraldsson[§], Kaisa M. Linderborg[†,*]

[†] Food Sciences, Department of Life Technologies, University of Turku, FI-20014 Turku, Finland

[‡] Faculty of Pharmaceutical Sciences, University of Iceland, IS-107 Reykjavík, Iceland

[§] Faculty of Physical Sciences, University of Iceland, IS-107 Reykjavík, Iceland

*corresponding author:

Professor Kaisa M. Linderborg

Food Sciences Unit, Department of Life Technologies

University of Turku, FI-20014 Turku, Finland

email: kaisa.linderborg@utu.fi; phone: + 358 50 439 5535

Supplementary Text 1. Identification of undigested compounds and digestion and oxidation products.

Identification of undigested lipids and digestion compounds. The AAB- and ABA-type TAGs were identified by the neutral loss of the PUFA (*m/z* 551.5), neutral loss of 16:0 (*m/z* 623.5 and *m/z* 597.5 for DHA and EPA, respectively) and 16:0 acyl chain (*m/z* 313.3), while DHA-TAG was identified by the neutral loss of DHA (*m/z* 695.5) and by the fragment *m/z* 385.3 (DHA acyl chain + 74). On the other hand, DHA-EE was confirmed by DHA acyl chain fragment (*m/z* 311.2) and DHA acyl chain with loss of water (*m/z* 293.2) (**Table 1**, main text). After digestion of structured lipids, identified DAGs were 16:0/16:0 and 16:0/PUFA. The earlier was identified also in undigested samples, likely as remainder from the synthetic pathway (see Section 2.2. of main text). The fragments *m/z* 313.3 (loss of 16:0 and ammonia), *m/z* 551.5 (precursor ion with loss of water), and *m/z* 339.3 (loss of free glycerol position and acyl chain fragmentation) allowed the identification of DAG 16:0/16:0. The DAG containing DHA was identified by the loss of DHA and ammonia (*m/z* 313.3), loss of 16:0 and ammonia (*m/z* 385.3), and by the precursor ion (*m/z* 641.5). The DAG containing EPA was identified as well by the losses of ammonia with 20:5 or 16:0 (*m/z* 313.3 and *m/z* 359.3, respectively). In DHA-TAG samples, DAG 22:6/22:6 was found in digestates and, in small amounts, in undigested sample. It was identified by the fragments *m/z* 385.3 (neutral loss of DHA and ammonia), *m/z* 311.2 (DHA acyl chain), and *m/z* 293.2 (DHA acyl chain with loss of water) (**Table 1**, main text). Monoacylglycerols were identified in all triacylglycerols digestates. In all structural lipids digestates, MAG 16:0 was found. It was identified with the fragments *m/z* 335.3 (protonated precursor ion), 226.2 (16:0 acyl chain with loss of water), and *m/z* 263.2 (sodiated 16:0). Noticeably, only structured lipids with PUFA in *sn*-2 position produced MAG 22:6 or MAG 20:5. The earlier was identified with the fragments *m/z* 311.2 (DHA acyl chain), *m/z* 385.3 (neutral loss of DHA and ammonia), and *m/z* 403.3 (protonated precursor

ion), while the latter produced the fragments *m/z* 267.2 (EPA acyl chain with loss of water), *m/z* 285.2 (EPA acyl chain), and *m/z* 303.2 (protonated EPA).

Identification of oxygenated compounds. Except for MAGs, of which no oxygenated species were identified, molecules with the addition of one oxygen were observed. This oxygen was present as M + 16, which could be ascribed to an epoxyde replacing a double bond or to an hydroxyl group, and as M + 14, which could be ascribed to a ketone or to an epoxyde adjacent to a double bond [1]. The M + 16 and M + 14 compounds were observed for all triacylglycerols, with only DHA-TAG lacking TAG + 14. DAGs containing PUFAs were found to be present also with the addition of one oxygen. DAG 16:0/22:6 was found in M + 16 and M + 14 form. The DAG 16:0/20:5 was found in M + 16 form, while DAG 16:0/20:5 + 14 was not detected. No DAG with addition of single oxygen was found after DHA-TAG digestion. At the same time, there was no detectable fragmentation for DHA-TAG + 16. Also, no species with single oxygen were found in DHA-EE (**Table 1**, main text). The main fragment identifying DAG + 16/14 was the sodiated PUFA + 16/14 Da, i.e. *m/z* 367.2/365.2 for DHA and *m/z* 341.2/339.2 for EPA.

Molecules with the addition of two oxygens were observed prior and after digestion. Molecules with two oxygens produced fragments ascribable to different isomers, as PUFAs contain multiple abstractable hydrogens and consequently different electron-deficient chain positions favoring reaction with oxygen[2]. The observed fragments for TAG 16:0/16:0/22:6 + 32 were ascribable to 20-OOH (*m/z* 887.7), 16-OOH (*m/z* 806.6), and 17-OOH (*m/z* 847.7). The fragment *m/z* 383.2 (sodiated DHA + 32) further confirmed the assignment[3]. In the case of TAG 16:0/16:0/20:5, the observed fragment *m/z* 781.5 was ascribable to 14-OOH isomer. The main fragments of DHA-TAG + 32 were *m/z* 383.2 (sodiated DHA + 32), *m/z* 950.6 (loss of acyl chain ascribable to 14-OOH), and *m/z* 991.6 (ascribable to 17-OOH). Also DHA-EE + 32 was observed. Its fragments *m/z* 365.2, *m/z* 285.1, and *m/z* 245.1 were ascribable to 20-

OOH (loss of 46 Da) and 14-OOH (loss of 126 Da), and 11-OOH (loss of 166 Da), respectively[4]. Diacylglycerols with addition of two oxygens produced ion currents but no fragments were observed and results therefore are based solely on peak integration. Likewise, no MS/MS fragmentation of triacylglycerols added with four oxygens was observed (**Table 1**, main text).

References.

(1) Xia, W.; Budge, S. M. Techniques for the Analysis of Minor Lipid Oxidation Products Derived from Triacylglycerols: Epoxides, Alcohols, and Ketones. *Compr. Rev. Food Sci. Food Saf.* **2017**, *16* (4), 735–758. https://doi.org/10.1111/1541-4337.12276.

(2) Schaich, K. M.; Shahidi, F.; Zhong, Y.; Eskin, N. A. M. Lipid Oxidation. In *Biochemistry of Foods*; Elsevier, 2013; pp 419–478. https://doi.org/10.1016/B978-0-08-091809-9.00011-X.

(3) Damerau, A.; Ahonen, E.; Kortesniemi, M.; Gudmundsson, H. G.; Yang, B.; Haraldsson, G. G.; Linderborg, K. M. Docosahexaenoic Acid in Regio- and Enantiopure Triacylglycerols: Oxidative Stability and Influence of Chiral Antioxidant. *Food Chem.* **2022**, 134271. https://doi.org/10.1016/J.FOODCHEM.2022.134271.

(4) Ahonen, E.; Damerau, A.; Suomela, J.-P.; Kortesniemi, M.; Linderborg, K. M. Oxidative Stability, Oxidation Pattern and α-Tocopherol Response of Docosahexaenoic Acid (DHA, 22:6n–3)-Containing Triacylglycerols and Ethyl Esters. *Food Chem.* **2022**, *387* (March), 132882. https://doi.org/10.1016/j.foodchem.2022.132882.

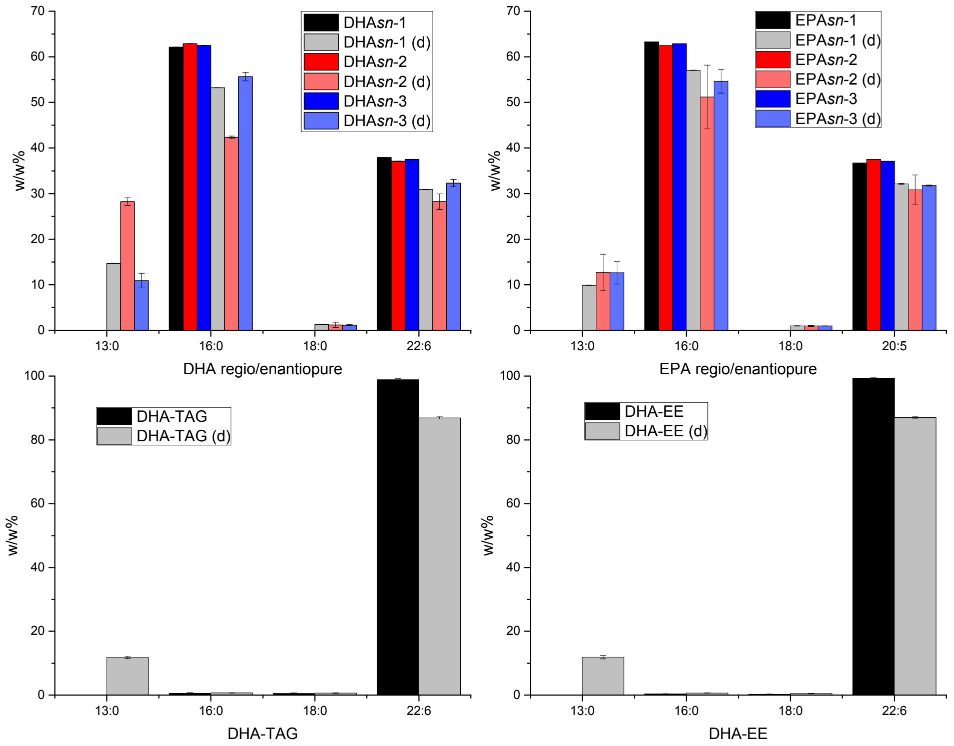

Supplementary Figure 1. Fatty acid composition, reported as w/w%, of regio- and enantiopure triacylglycerols, DHA-TAG (docosahexaenoin), and DHA-EE (ethyl docosahexaenoate). Digestates are marked with (d). Fatty acid 13:0 is tentatively assigned.

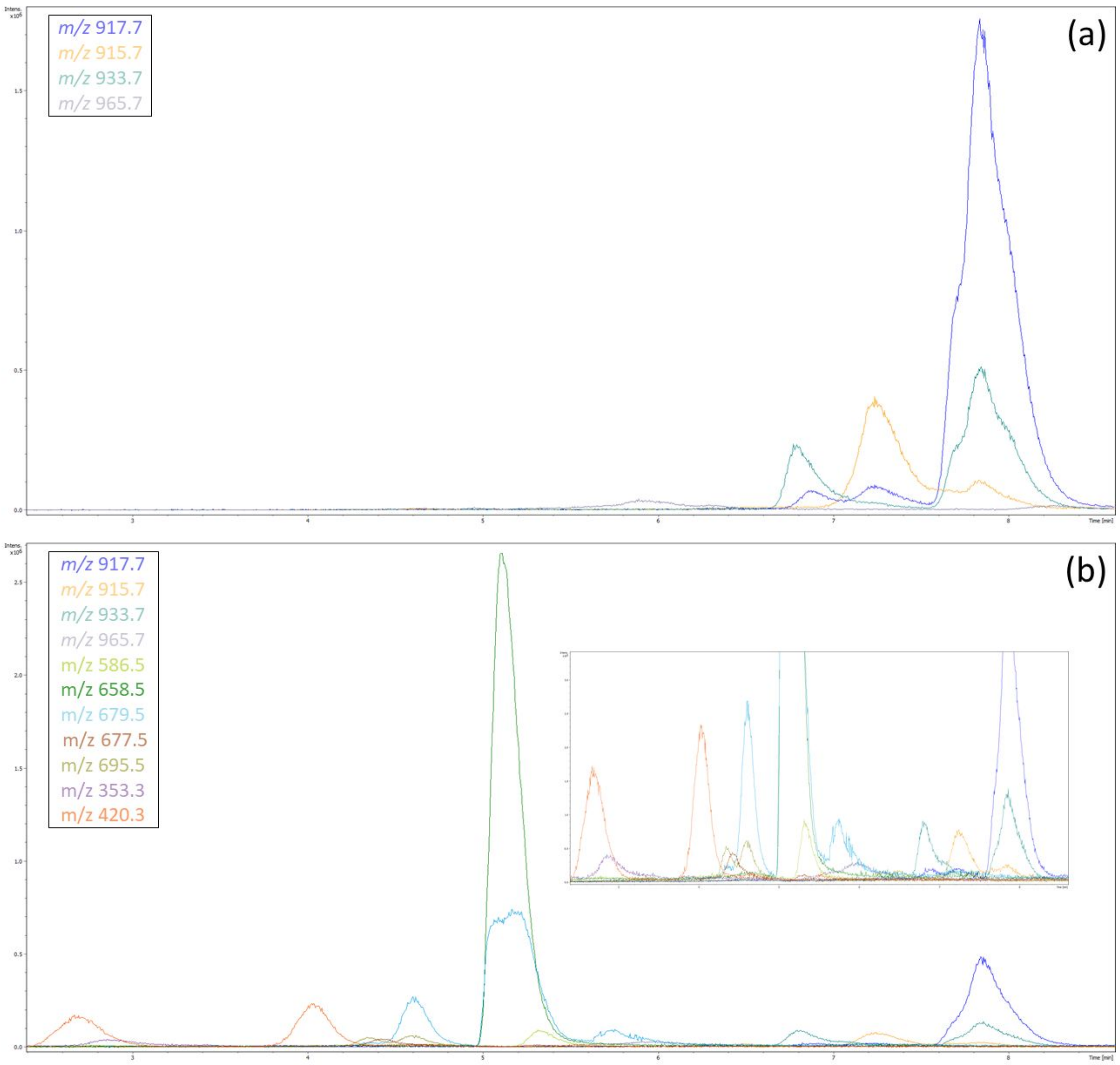

Supplementary Figure 2. Ion currents of DHA*sn*-2 before (a) and after (b) digestion. The ion currents are produced by: [TAG 16:0/16:0/22:6 + 16 + Na]$^+$ (*m/z* 917.7); [TAG 16:0/16:0/22:6 + 14 + Na]$^+$ (*m/z* 915.7); [TAG 16:0/16:0/22:6 + 32 + Na]$^+$ (*m/z* 933.7); [TAG 16:0/16:0/22:6 + 64 + Na]$^+$ (*m/z* 965.7); [DAG 16:0/16:0 + NH_4]$^+$ (*m/z* 586.5); [DAG 16:0/22:6 + NH_4]$^+$ (*m/z* 658.5); [DAG 16:0/22:6 + 16 + Na]$^+$ (*m/z* 679.5); [DAG 16:0/22:6 + 14 + Na]$^+$ (*m/z* 677.5); [DAG 16:0/22:6 + 32 + Na]$^+$ (*m/z* 695.5); [MAG 16:0 + Na]$^+$ (*m/z* 353.3); [MAG 22:6 + NH_4]$^+$ (*m/z* 420.3).

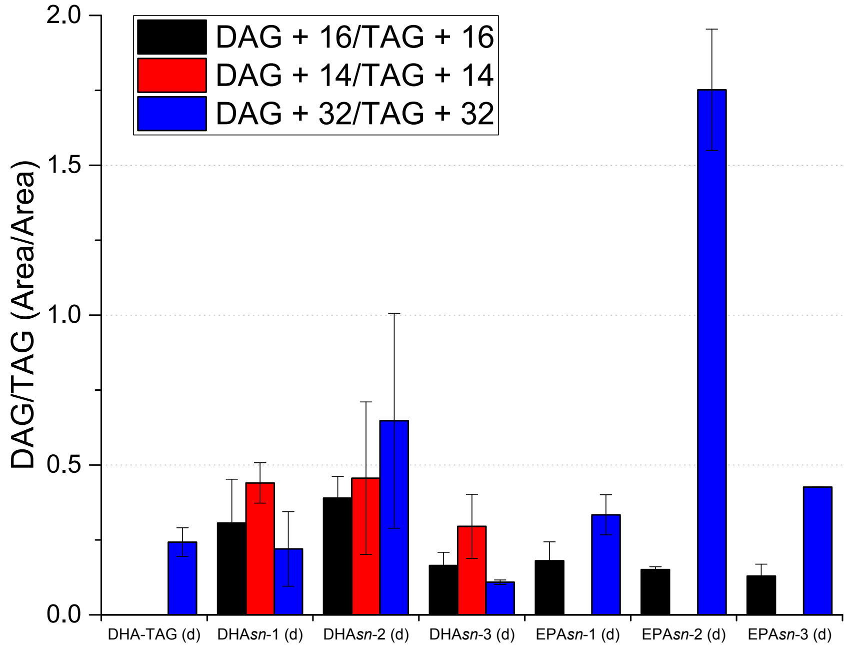

Supplementary Figure 3. Ratio between oxygenated DAGs and TAGs in digestates of ABA and AAB-type TAGs and DHA-TAG.

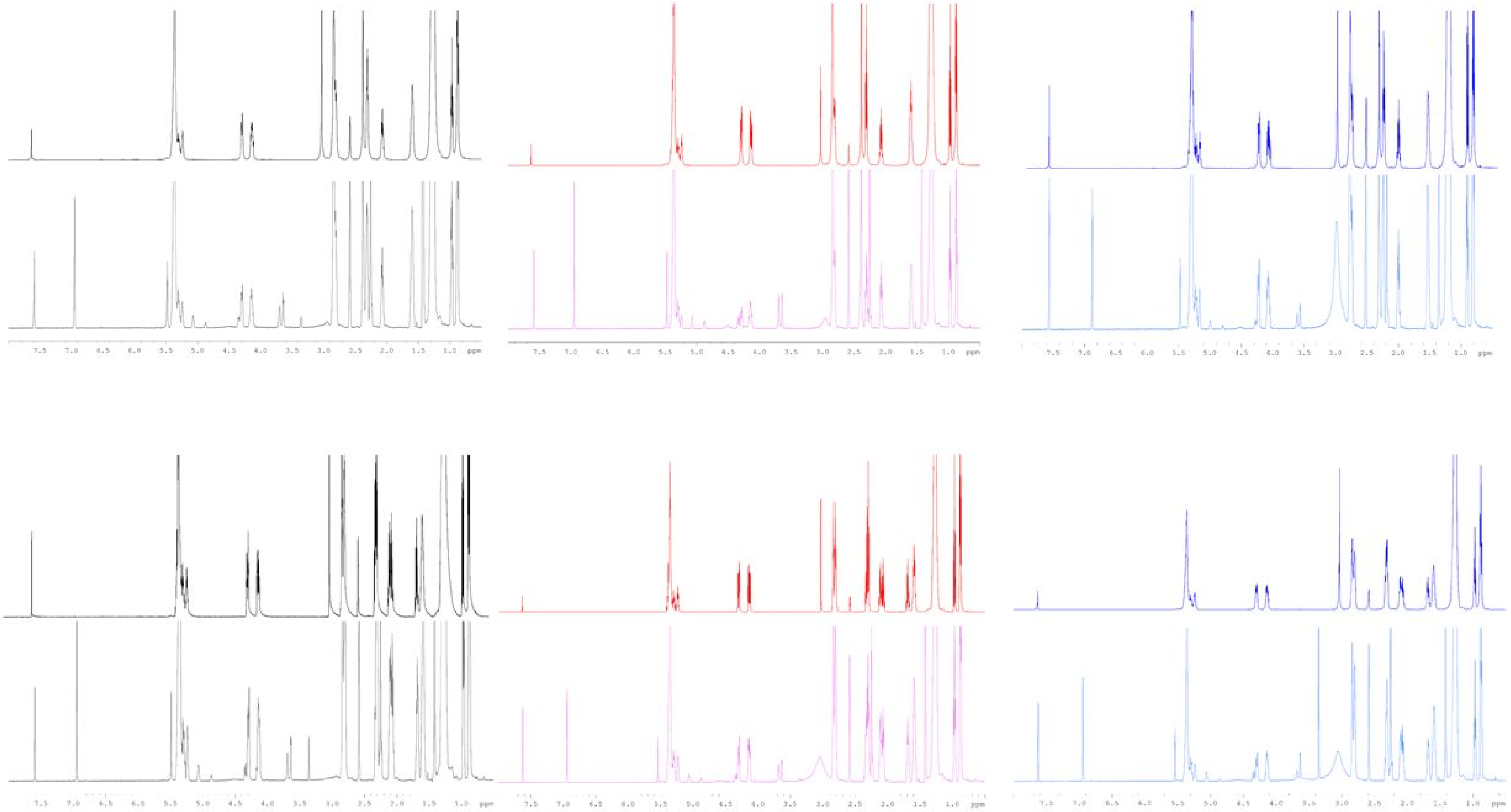

Supplementary Figure 4. ^{1}H NMR spectra of regio- and enantiopure triacylglycerols. From left to right, from top to bottom: DHA*sn*-1, DHA*sn*-2, DHA*sn*-3, EPA*sn*-1, EPA*sn*-2, and EPA*sn*-3. The colors black, red, and blue mark *sn*-1, *sn*-2, *sn*-3, respectively. Spectra in lighter colors are obtained from the respective digestates. All spectra are recorded in $CDCl_3$:DMSO-d6 5:1 at 298 K.

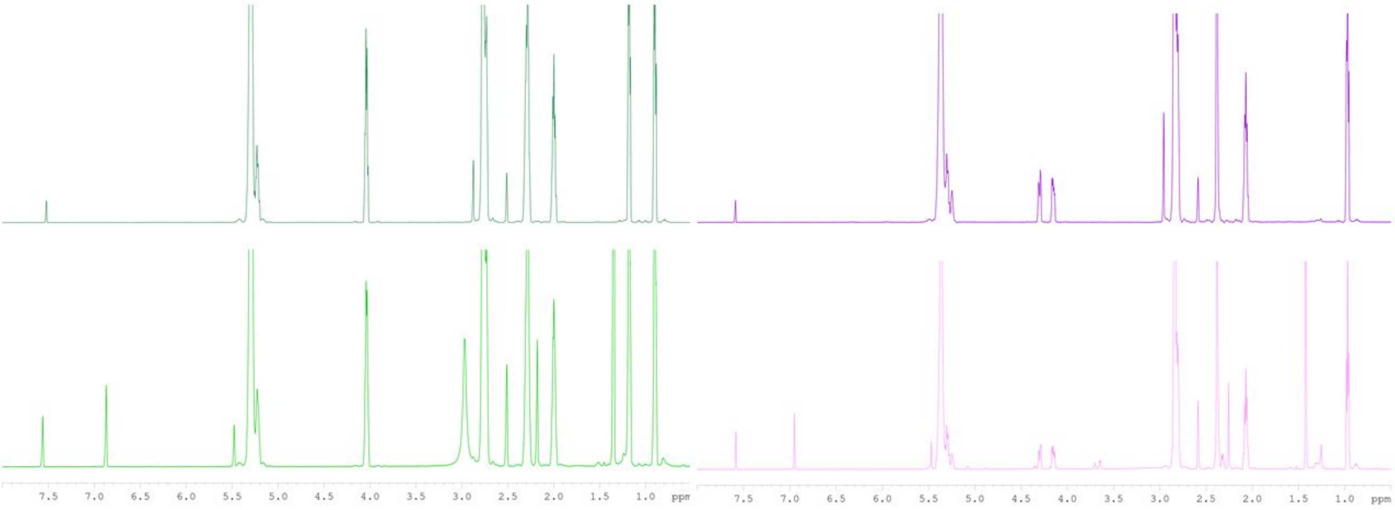

Supplementary Figure 5. ^{1}H NMR spectra of DHA-EE (left) and DHA-TAG (right). Spectra in lighter colors are obtained from the respective digestates. All spectra are recorded in $CDCl_3$:DMSO-d6 5:1 at 298 K.

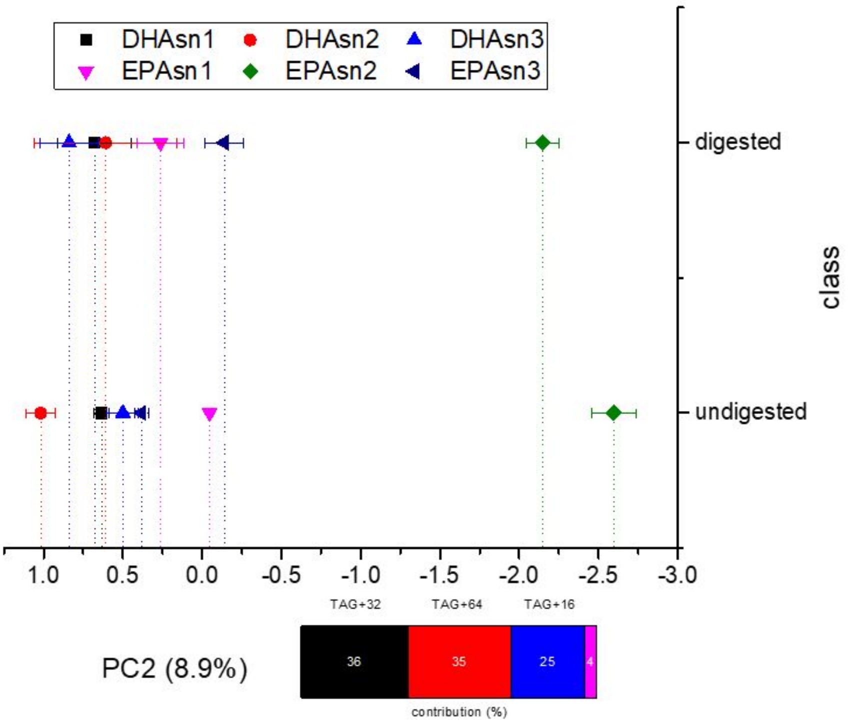

Supplementary Figure 6. Average score values on the second principal component of the PCA model computed with HPLC-qTOF data of structural lipids after removal of MAG PUFA. Main contributors of PC2 variance are reported in the bar plot below the x-axis.

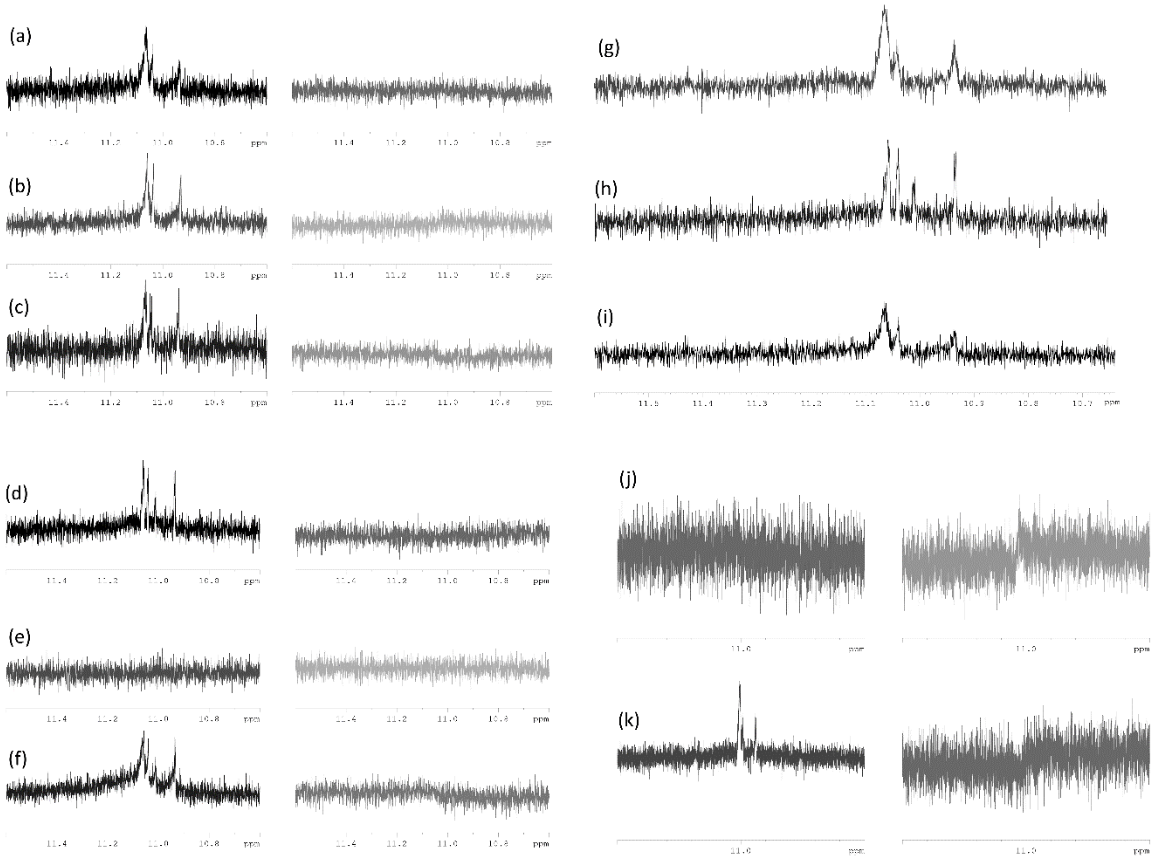

Supplementary Figure 7. Selective gradient excitation ^{1}H NMR spectra (hydroperoxide region) prior and after simulated digestion. Left column shows ABA- and AAB-type structures: DHA*sn*-1 (a), DHA*sn*-2 (b), DHA*sn*-3 (c), EPA*sn*-1 (d), EPA*sn*-2 (e), EPA*sn*-3 (f). Upper right column shows magnified spectra of, from top to bottom, DHA-TAG (g), EPA*sn*-1 (h), and DHA*sn*-1 (i). Lower right column shows DHA-EE (j) and DHA-TAG (k).
